# Radioguided Surgery for Gastroenteropancreatic Neuroendocrine Tumours: a Systematic Literature Review

**DOI:** 10.1007/s11605-021-05115-w

**Published:** 2021-09-10

**Authors:** Katrina Clair Cockburn, Zaher Toumi, Alison Mackie, Peter Julyan

**Affiliations:** 1 Northern Medical Physics and Clinical Engineering, County Durham and Darlington NHS Foundation Trust, Hollyhurst Road, Darlington, DL3 6HX UK; 2 Department of General Surgery, County Durham and Darlington NHS Foundation Trust, Hollyhurst Road, Darlington, DL3 6HX UK; 3 Department of Nuclear Medicine, The Christie Hospital NHS Foundation Trust, Manchester, UK

**Keywords:** Radiopharmaceutical, Neuroendocrine tumours/surgery, Diagnostic techniques, Radioisotope

## Abstract

**Background:**

Radioguided surgery (RGS) for gastroenteropancreatic neuroendocrine tumours (GEP-NETs) has been suggested as a way to improve intraoperative lesion detection. This systematic literature review of reports of the use of RGS for GEP-NETs was performed to determine if there is a benefit.

**Methods:**

A literature search was conducted using Google Scholar and PubMed, and snowballing from any relevant literature. Full-text studies were included if they were published in the English language and reported outcomes of RGS on human subjects with GEP-NETs. Qualitative data synthesis was performed.

**Results:**

Twenty-six papers including a total of 209 patients were included. The tracers used were predominantly indium-111 pentetreotide, gallium-68 DOTA-peptides, and technetium-99m EDDA/HYNIC-peptides. Heterogeneous protocols make comparisons difficult, but most papers reported a benefit from the use of RGS in tumours in the gastrointestinal tract; utility in localisation of pancreatic tumours was less clear. Time between tracer administration and operation varied: from 16 h to 8 days with indium-111, 0–24 h with technetium-99m, and 19–193 min with gallium-68. Eight teams reported the thresholding technique used for discrimination—four used a ratio, four statistical methods, and one looked at the sensitivity and specificity of different cut-offs. Six teams performed follow-up of 24 patients (three pancreas, eight gastrinoma, 13 gastrointestinal tract) for between 3 months and 3 years. Two patients relapsed (one pancreas, one gastrinoma) between 6 and 12 months post-surgery.

**Conclusions:**

RGS appears to aid in localisation of gastrointestinal NETs, but the benefit is more equivocal in pancreatic NETs. Further work into outcomes is warranted.

## Introduction

Gastroenteropancreatic neuroendocrine tumours (GEP-NETs) account for only 2% of gastrointestinal tract tumours; however, their prevalence is increasing, with a reported four-fold increase in reported cases since the 1980s ^1,2^.

Whilst many reviews state that GEP-NETs are indolent, this view has been challenged by better epidemiological data: The 2011 National Cancer Institute Surveillance, Epidemiology and End Results (SEER) Registry report of the epidemiology of GEP-NETs suggests that the overall 5-year survival for patients is between 60 and 70%, being 88% for patients with rectal NETs, but only 37% for functional pancreatic NETs ^3^.

Surgery remains the primary intervention for GEP-NETs with the aim of radical treatment if possible, but debulking to minimise side effects might be performed if there is evidence of metastatic disease ^4^. The silent presentation and spread of the primary tumour may result in high recurrence rates; it is estimated that up to 30% of laparotomies fail to sterilise the tumour bed and hence control the disease ^5,6^. This high failure rate undoubtedly contributes to unfavourable outcomes for patients.

The ability to more accurately identify and localise tumours has the potential to reduce the number of failed laparotomies. Reports in the literature suggest that radioguided surgery (RGS) may aid surgeons in discriminating between cancerous and non-cancerous tissues, as well as localising occult, impalpable tumour deposits which may otherwise be missed.

In radioguided surgery, the surgeon uses a hand-held radiation detector to localise tissues which have been “tagged” with a radioactive material. This technique has historically been used to identify occult or impalpable lesions, to map the lymphatic basin to which tumours drain, or to verify clear surgical margins. In order to label neuroendocrine tumours, the radiopharmaceuticals that are chosen either bind to receptors on the cell surface or are taken up by active means and incorporated into the processes of the cell itself ^7^.

Typically 80–100% of GEP-NETs overexpress five different subtypes of somatostatin receptors (SSTRs), with type 2 being the most commonly found in all NETs ^8^. Synthetic somatostatin analogues (SSAs) labelled with short-acting radioactive tracers which were initially developed for imaging can be employed as the tracer for RGS for GEP-NETs. Whilst natural somatostatin binds to all receptor subtypes, SSTR 2 and to a lesser degree SSTR 5 demonstrate the greatest affinity for somatostatin analogues, whilst SSTRs 1, 3, and 4 show minimal affinity for any synthetic somatostatin ^8^.

Whilst SSA tracers will bind to many NETs, a sub-group does not demonstrate increased SSTR density and require a different tracer. Meta-iodobenzylguanidine (mIBG) is an analogue of guanethidine that is used in the production of catecholamines and so becomes stored in the vesicles of hypersecretory adrenal medullary and medullary thyroid tumours ^7^. Iodination with radioiodines allows mIBG to act as a radiolabelled tracer for radioguided surgery.

The radiation detectors used in RGS are typically hand-held probes with a means of detecting the radiation at the tip; for most commercially available probes, this is a scintillation crystal surrounded by lead collimation with a radiolucent window. The signal produced by the probe is fed back to the surgeon by means of an auditory and/or visual signal representing the count rate at the tip of the probe. Modern probes allow the user to select an “energy window” which permits the surgeon to discriminate between gamma-emitters with different energies. Gamma probes may also be used with positron emitting radiopharmaceuticals. However, as the 511 keV annihilation photons are more penetrative than those from gamma-emitting tracers, the probes require thicker collimation to maintain the directional information.

The objective of this systematic literature review is to examine the evidence for the use of RGS in treatment of GEP-NETs and to identify any gaps in the knowledge base.

## Materials and Methods

Following the PRISMA methodology ^9^, a search using PubMed and Google Scholar was performed using the following search terms:

- “Radioguided” AND “Surgery” AND “Neuroendocrine” AND “Tumours”, OR “Radioguided” AND “Surgery” AND “Neuroendocrine” AND “Neoplasm”, OR “Radioguided” AND “Surgery” AND “Tektrotyd”, OR “Radioguided” AND “Surgery” AND “Somatostatin”, OR “NETs” AND “Radioguided”

The searches were not limited to a specific date range, but were restricted to full-text publications in the English language which reported the outcomes of radioguided surgery using cell-marking radioactive tracers on human subjects with GEP-NETs. Where papers included multiple tumour types or other techniques but adequately described the outcomes of the patients to allow these to be excluded, these patients were included. Exclusion criteria were meta-analyses, reviews, and patients with pathologies other than GEP-NETs.

Data extraction was performed by the lead author. Risk of bias assessment was not performed as most of the papers were either individual case reports or small case series with no comparator and therefore are intrinsically at increased risk of bias.

## Results

### Papers Selection and Systematic Literature Review

**Fig. 1:**
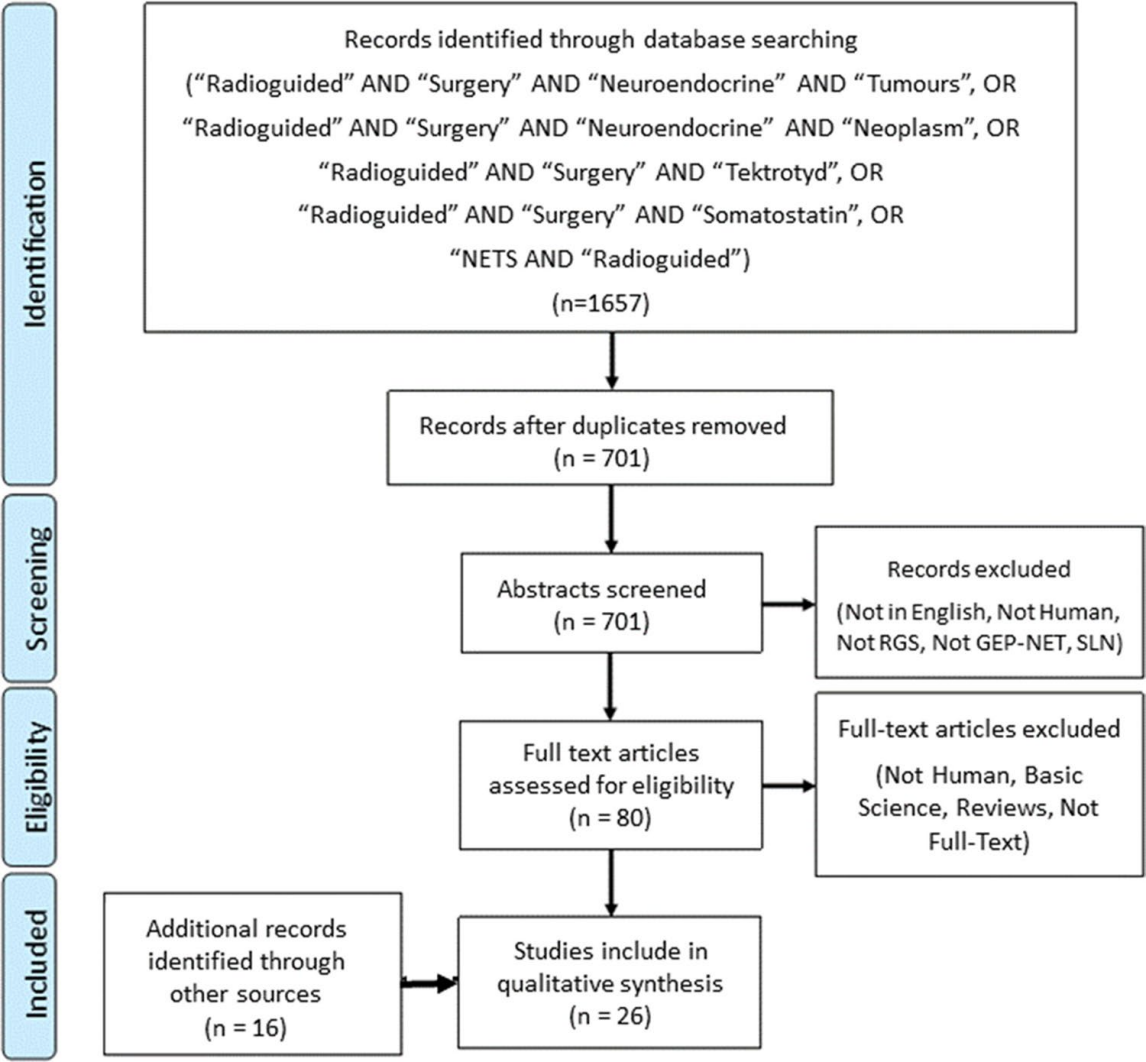
PRISMA flow diagram for literature search

The literature search led to the identification of 1657 records (Fig. 1) which included 701 unique papers which met the identified search criteria. After abstract review, 621 papers were excluded. Cross-reference check provided 16 more articles to review. Assessment of full-text led to the inclusion of 26 papers.

### Study Characteristics

The 26 papers included a total of 209 patients with GEP-NETs who underwent RGS. In 11 papers (93 patients), the tracer was indium-111 pentetreotide (OctreoScan™) with one of these papers also using iodine-123 labelled mIBG in a separate set of six patients. Six papers (67 patients) described the use of gallium-68 labelled DOTA-TATE or DOTA-TOC, five papers (39 patients) used a technetium-99m labelled SSA, and the remaining four papers (four patients) described the use of a variety of other tracers. The earliest paper was published in 1996, the most recent in 2019.

### RGS with Indium-111 Pentetreotide (OCT)

Of the 93 patients included, 2 had carcinoid tumours located in the stomach, 8 had gastrinomas located in the duodenum (including 6 patients with Zollinger-Ellison Syndrome), 66 had mid-gut carcinoids (MGC), two had rectal carcinoids, and 15 had pancreatic tumours (including insulinoma) (Table 1). Due to the different reporting styles, combining the data from these studies is difficult; however, three themes emerged:

- In all reported cases of tumours in the stomach, duodenum, mid gut, and rectum, the use of the gamma probe aided in identification of primary and nodal metastases over localisation by conventional imaging such as CT or MRI, somatostatin receptor scintigraphy (SRS) with OCT, or standard operative techniques, in some instances demonstrating occult lesions not otherwise demonstrated.
- Where reported, the gain in sensitivity in gastric tract tumours does not imply poor specificity; the results of Wängberg et al. and Öhrvall et al. imply a combined sensitivity of 86% with a specificity of 80% ^18,20^.
- The case for RGS for pancreatic disease is less clear-cut; whilst most reports demonstrated a benefit, three of the four cases of endocrine pancreatic tumours reported by Wängberg et al. had failed RGS explorations, with the fourth only demonstrating one lesion ^20^.

**Table 1:** Summary of published studies examining the use of OCT for radioguided surgery

Author	Ref	Number of cases	Tumour type	Tumour location	Peptide mass (µg)	Activity (MBq)	Delay	Results	Comments
Hall et al	^10^	6	ZES	Duodenum	N/S	229.6 (± 15.2)	24 h	Identified additional foci in 3/6 cases	Also used intraoperative gamma camera
Rossetti et al	^11^	1	Rectal carcinoma	Rectum	N/S	500	16 h	Identified 15 mm node in iliac fossa with TBR of 7:1	
Wang et al	^12^	30	MGC	Midgut	N/S	203.5 (range 37–276)	1–8 d	29/30 probe was "helpful" or "essential"	Wide range of protocols due to referral process. A 3- to 7-day surgery–injection interval with the injection of 148–259 MBq of ^111^In.pentetreotide appeared to be optimal
Hosoya et al	^13^	1	Gastric carcinoid	Stomach	N/S	N/S	20 h	Identified both primaries, no uptake found in pancreas or surrounding tissue	
Banzo et al	^14^	1	Rectal carcinoid	Rectum	N/S	111	24 h	Identified nodal metastasis missed at previous surgery	
Pelaez et al	^15^	1	Insulinoma	Pancreas	N/S	N/S	48 h	Identified nodal metastasis	Relapsed after previous surgery
Albertario et al	^16^	1	Gastrinoma	Duodenum	N/S	120	20 h	Identified primary and 10 mm nodal metastasis	Technique adopted as patient obese
Benjegård et al	^17^	6	MGC	Midgut	10–20	200–260	1–7 d (mean 4)	11/12 correctly identified	TBR Threshold based on 2SD with time set to acquire > 100 background counts
		1	EPT	Pancreas			1 d	1/1 correctly identified	
Öhrvall et al	^18^	13	MGC	Midgut	20	108–194	24–48 h	32 TP, 8 TN 1 FP, 3 FN (all < 5 mm)	All TP lesions had TBR 1.4 or greater
		8	Pancreas	Pancreas					
Adams et al	^19^	10	Carcinoid	Midgut	10–20	110–220 (mean 180)	24 h	Probe 70/70 (no FP) Palpation 31/70 Conventional Imaging 30/70 SRS 52/70	
		1	Gastrinoma	Duodenum					
		1	Insulinoma	Pancreas					
Wängberg et al	^20^	7	MGC	Midgut	10–20	140–300	24–168 h	25 TP, 0 TN, 1 FP, 4 FN	
		1	Gastric carcinoid	Stomach			24 h	2 TP, 0 TN, 0 FP, 0 FN,	
		4	EPT	Pancreas			48–120 h	1 TP, 0 TN, 0 FP, 5 FN	One not determined, one failed probe localisation

*ZES* Zollinger-Ellison syndrome, *MGC* midgut carcinoma, *N/S* not stated, *LFoV* large field of view, *TP* true positive, *TN* true negative, *FP* false positive, *FN* false negative, *EPT* endocrine pancreatic tumour, *TBR* tumour to background ratio, *SRS* somatostatin receptor scintigraphy

Two groups attempted to determine the best interval between administration and surgery. Wang et al. used retrospective data from surgical records to identify the interval which leads to the best target to background ratio (TBR) following administration of between 37 and 277 MBq of OCT in referring hospitals between 1 and 8 days before surgery. Their findings were that the peak TBR occurred 5 days after administration of 150–260 MBq, but that renal activity at that point was still significant and could mask small lesions. Their recommendation was to perform surgery at 7 days post administration ^12^.

Wängberg et al., however, performed a prospective study, performing surgery at between 1 and 7 days post administration of between 140 and 300 MBq of OCT ^20^. Unlike Wang, Wängberg found that there was little difference in the TBR for resected tissues over the five time points on which imaging occurred, and the study did not identify the optimal interval. The majority of published studies using OCT described a 24–72-h interval between administration and surgery. Even though this is a much shorter period than suggested as optimal by Wang, 10 of the 11 authors described how RGS benefitted the surgeon by identifying additional foci over other techniques ^10–12,15,16,18,19^; minimising the extent or duration of surgery ^12–14,16^; assisting with differentiation between normal tissue and tumour ^12,13,15^; and /or aiding the surgeon in localising the tumour ^12,15–17^.

### RGS with Technetium-99m Labelled Tracers

OCT is well established, but has significant drawbacks for imaging and RGS. The long half-life and polychromatic energy emissions of indium-111 leads to a high radiation dose; the International Commission on Radiation Protection Publication 128 lists the effective dose as 12 mSv for an administered activity of 220 MBq ^21^. Whilst this dose can be justified on the basis that SRS and RGS can aid in the diagnosis and treatment of GEP-NETs, the shift to radical treatment for this patient population suggests that imaging radiation doses are likely to become an increasing concern.

The higher energy emissions from indium-111 also lead to problems with imaging and in vivo detection with a gamma probe. Gamma cameras and probes are optimised for the 140 keV peak from technetium-99m; the higher energy 173 and 247 keV peaks from indium create problems with collimation and shielding, leading to increased background, scattered photons, and reduced spatial resolution.

The final problem with OCT is that it has to be ordered from the manufacturer specifically for the patient; with a lead time of several days, this can lead to delays in diagnosis and treatment.

A range of somatostatin analogues labelled with technetium-99m have been devised, and with the favourable emissions for imaging and RGS, they have the potential to overcome many of the problems with OCT. The two most widely used technetium-99m labelled somatostatin analogues, ^99m^Tc.EDDA/HYNIC-TOC (TOC) and the biologically similar ^99m^Tc.EDDA/HYNIC-Octreotate (TATE), have also been trialled in RGS (Table 2).

**Table 2:** Summary of published studies examining the use of TOC for radioguided surgery

Author	Ref	Number of cases	Tumour type	Tumour location	R’pharm	Peptide mass (ug)	Activity (MBq)	Delay	Results	Comments
Maccauro et al	^22^	3	MGC	Midgut	TOC	N/S	185	4 h	All primaries identified and excised Nodes: TP 35, TN 72, FP 0, FN 0	Includes detection of an extra-regional node
		1	Rectal	Rectal						
		1	Gastric NEC	Stomach						
Hodolič et al	^23^	5	MGC	Midgut	TOC	20*	550–650	3–6 h	Identified 5/5	*Amount of peptide prepared 11/16 tumours located in pancreas on imaging
		4	Insulinoma	Pancreas					Identified 2/4	
		3	Gastrinoma	Pancreas					Identified 1/3	
		4	NEC	Pancreas					Identified 3/4	
Hubalewska-Dydejczyk et al	^24^	5	Carcinoid	Not stated	TATE	10	700	24 h	Not stated	
Hubalewska-Dydejczyk et al	^25^	4	Carcinoid	Midgut	TATE	10	700	24 h	RGS: 3/3 tumours, 7/8 nodes, 1 FP SRS: 3/3 tumours, 3/8 nodes, 1FP	Peptide dose based on administration of half of 20 ug of peptide in kit
		5	Pancreatic	Pancreas					RGS: 5/5 tumours SRS: 5/5 tumours	
Fettich et al	^26^	2	Gastrinoma	Not Stated	TOC	10	600	4 h	Identified all tumours with TBR of > 3	
		1	Insulinoma	Pancreas						
		1	Carcinoid	Not stated						

*MGC* midgut carcinoma, *NEC* neuroendocrine carcinoma, *TOC* ^99m^Tc.EDDA/HYNIC-TOC, *TATE* ^99m^Tc.EDDA/HYNIC-octreotate, *N/S* not stated, *RGS* radioguided surgery, *TP* true positive, *TN* true negative, *FP* false positive, *FN* false negative

Maccauro et al. administered 185 MBq of TOC to five patients (3 with midgut carcinoid, 1 gastric neuroendocrine carcinoma (NEC), 1 insulinoma) 4 h prior to surgery. The patients all underwent probe guided tumour and nodal excision, followed by regional lymphadenectomy. The probe aided in the excision of all primary tumours and had 100% sensitivity and specificity for nodal disease, including identifying an extra-regional nodal metastasis which would not otherwise have been removed ^22^.

Hodolič et al. reported on a series of 16 patients with 17 tumours, comprised of 5 MGCs and 12 pancreatic neuroendocrine tumours (pNETs) including 4 insulinomas, 2 gastrinomas, 3 NECs, and 3 pancreatic tumours of unknown aetiology ^23^. The investigators took a threshold of 4:1 as the cut-off TBR value, and 3–5 h following administration of 550–650 MBq of TOC to the patients performed 12 successful laparotomies, with five failures (one of which was later found to have negative SRS follow-up imaging).

Fettich et al. found better results with TOC for RGS, reporting on its use in four patients; one with a carcinoid tumour, one with an insulinoma, and two with gastrinoma ^26^. Four hours after administration of 600 MBq of TOC, all lesions found scintigraphically were identified with the gamma probe, with TBR values in excess of 3:1. Ex vivo imaging and count rates confirmed the excision of the tumour tissues, and all patients remained clinically, scintigraphically, and biochemically free of disease 3 months after surgery.

Finally, Hubalewska-Dydejczyk et al. published two papers examining the use of TATE in RGS. The first, published in 2006, reported on 5 patients with carcinoid tumours in unspecified locations. The second paper in 2007 reported the results of RGS performed in 4 patients with MGCs, and 9 with pNETs (4 insulinoma and 5 glucagonoma) ^24,25^. Hubalewska-Dydejczyk found the use of TATE to be beneficial in all cases reported in the 2006 paper, reporting that in 2 of the 5 procedures, RGS made intraoperative identification and resection of the primary tumour possible. The 2007 paper also found RGS to be of benefit; RGS identified all five pancreatic tumours, all three carcinoid tumours, and 7 of 8 nodal metastases, whereas SRS only identified 3 of the 8 nodes. However, they did have one false positive result as a result of increased tracer uptake due to Crohn’s disease.

### RGS with Other Tracers

OCT and TOC both have a long pedigree for use in RGS; however, authors have also tried using alternative tracers (Table 3). Five groups—El Lakis, Kunikowska, Sadowski, Kaemmerer, and Freesmeyer—have published a total of 6 papers, looking at RGS for GEP-NETs using positron emitting gallium-68-DOTA-peptides ^27–29,31,32^.

**Table 3:** Summary of published studies examining the use of other non-^99m^Tc radiopharmaceuticals for radioguided surgery

Author	Ref	No of cases	Tumour type	Tumour location	R’pharm	Peptide mass (ug)	Activity (MBq)	Delay	Results	Comments
El Lakis et al	^27^	19	Pancreatic	Pancreas	^68^Ga	N/S	185	None	Identified 133 lesions, 100 proven at histology 5 of 39 lymph nodes only detected with probe TBR of 2.5: sensitivity 90%, specificity 25% TBR of 16: sensitivity 54%, specificity 81% ROC AUC: 0.72	Changed background during trial which improved TBR results
		18	MGC	Midgut						
		4	Stomach	Stomach						
Kunikowska et al	^28^	1	MGC	Midgut	^68^Ga	N/S	80	N/S	Identified impalpable tumour in a Meckel’s diverticulum	
Sadowski et al	^29^	3	MGC	Midgut	^68^Ga	N/S	185	78.2 (10–193) min	Identified 44 lesions, 3 impalpable, 35 confirmed by pathology Correctly identified 81% of GIT tumours and nodes but only 66.7% of pNET	
		2	Gastrinoma	Duodenum (± stomach)						
		9	Pancreatic	Pancreas						
Todorović-Tirnanić et al	^30^	1	MGC	Midgut	^177^Lu ^90^Y	N/S	3000 6000	5 d	Identified all lesions seen on PET/CT scan, plus bilateral ovarian metastases	
Kaemmerer et al	^31^	7	Ileal NET	Midgut	^68^Ga	N/S	180	19–120 min	Probe 94% of lesions, PET/CT 69% and palpation, 50% RGS resulted in change in the operative procedure in 56%	
		1	Pancreatic	Pancreas						
		1	NET-CUP	Unknown						
Freesmeyer et al	^32^	1	Carcinoid	Duodenum	^68^Ga	N/S	N/S	N/S	Identified impalpable tumour allowing conservative surgery	
Yüksel et al	^33^	1	MGC	Midgut	mIBG	N/S	280	24 h	Identified 3 occult nodal metastases	
Benevento et al	^34^	1	MGC	Midgut	^125^I-OCT	10	19	Intra-op	Resected lesion	
Kunikowska et al	^35^	1	Pancreatic	Pancreas	^68^Ga	N/S	80	60 min	Not identified with probe	Used PET/CT of specimen to verify margins post-op
Arbizu et al	^36^	1	MGC	Midgut	^18^F-DOPA	N/S	N/S	N/S	Visually identified and confirmed with probe	
Wang et al	^12^	5	MGC	Midgut	mIBG	N/S	18.5–370	2–8 days	“Only helpful in one patient”	
		1	Pancreatic	Pancreas						

*GIT* gastrointestinal tract, *pNET* pancreatic neuroendocrine tumour, *NET-CUP* neuroendocrine cancer of unknown primary, ^*68*^*Ga* gallium-68 DOTA-peptides, ^*177*^*Lu* lutitium-177 DOTA-peptide, ^*90*^*Y* yttrium-90 DOTA-peptide, *mIBG* ^123^I-MIBG, *N/S* not stated

Case studies by Kunikowska and Freesmeyer both report the benefit of using RGS in identifying occult solitary lesions: Kunikowska describing a technically challenging localisation of a midgut carcinoma which was hidden in a Meckel’s diverticulum, and Freesmeyer reporting on the ability to change from the planned Whipple’s procedure to more conservative surgery on a patient with an impalpable carcinoid in the duodenum ^28,32^. The ability to locate impalpable and/or radio-occult lesions was also described in the three case series of El Lakis, Sadowski, and Kaemmerer ^27,29,31^. El Lakis reported that 5 of the 39 nodal metastases excised were only identified through the use of the probe; similarly, Sadowski reported identifying 3 out of 44 nodes only by use of the probe. Kaemmerer went further and compared the percentage of all excised lesions which were identified by different means; they reported that the probe identified 94% of tumours, pre-operative PET/CT 69%, and surgical palpation only 50%.

Whilst El Lakis and Kaemmerer gave pooled results, Sadowski broke down the results according to tumour location. In common with the findings of Wängberg who used OCT, Sadowski was also more successful at locating NETs in the gastrointestinal tract than in the pancreas (81% of gastrointestinal tract tumours, as opposed to 66.7% in the pancreas). In their 2014 paper, Kunikowska also reported difficulty in identifying a pancreatic tumour using RGS, resulting in the use of an en bloc resection, followed by a PET/CT scan of the specimen to verify the margins ^35^.

The success of RGS with ^68^Ga-DOTA-peptides despite the high energy of the annihilation photons is perhaps surprising—one of the perceived disadvantages of using OCT is the ability of the high energy indium-111 photons to penetrate the collimation of the probe and tissues, creating a high background count rate which may have confounded the TBR calculations. To negate the high background radiation levels from scattered gamma photons, the use of beta-emitting radiopharmaceuticals has been suggested as a way to improve the TBR during RGS, and this was attempted by Todorović-Tirnanić ^30^. The team looked at the success of RGS 5 days following administration of 3 GBq of yttrium-90-DOTA-TOC and 6 GBq of lutetium-177-DOTA-TOC. The beta-emitting therapy doses were administered as part of a fractionated course of molecular radiotherapy, and the authors were able to find MGCs less than 5 mm in diameter using beta probes; this compared well to the previously described studies which were only able to identify lesions in excess of 5 mm. The authors describe the low background present in the field due to the limited penetration of the beta-particles in tissue and postulate that this is what led to the superior resolution. The authors did not publish the extremity and whole-body doses of the staff involved in the procedure, nor the intent of performing the surgery mid-way through the course of radiotherapy.

Two groups, Yüksel and Wang, used iodine-123-mIBG to guide surgery in a total of 5 patients with MGCs and a single patient with a pNET ^12,33^. Yüksel et al. were successful in locating an MGC, despite the lower specificity of ^123^I-mIBG for neuroendocrine tumours than somatostatin analogues; however, Wang stated that RGS with ^123^I-mIBG was “only helpful in one patient”, implying that RGS failed to locate the tumours in the other five patients. Wang did not clarify where the tumour was located.

Finally, Benevento et al. and Arbizu et al. both published case studies regarding the use of RGS to localise MGCs, using iodine-125-octreotide and fluorine-18-DOPA, respectively ^34,36^. Both reported that the probe identified the tumours, with Benevento identifying an otherwise occult liver metastasis.

### Discrimination Between Normal and Target Tissues

Whilst many papers have not described the method by which they discriminated between normal and target tissue, the eight which did followed one of two protocols (Table 4). The simplest technique, which was adopted by four teams of investigators, was to set a threshold ratio which ranged from between 1.5 and 4 times the background count rate.

**Table 4:** Discrimination techniques and chosen thresholds

Author	Ref	Tracer	Thresholding technique	TBR threshold
Benevento et al	^34^	^125^I-OCT	Ratio	1.5:1
Sadowski et al	^29^	^68^Ga	Ratio	1.5:1
Adams et al	^19^	OCT	Ratio	2:1
Hodolič et al	^23^	TOC	Ratio	4:1
Benjegård et al	^17^	OCT	Statistical	2 standard deviations
Öhrvall et al	^18^	OCT	Statistical	2 standard deviations
Wängberg et al	^20^	OCT	Statistical	2 standard deviations
Hall et al	^10^	OCT	Statistical	3 standard deviations

All but one of the groups using the ratio technique found good results. Adams et al., in particular, found more than 70 histologically proven lesions with the probe with no false positives, compared to only 31 by palpation ^19^. Hodolič, however, failed to localise the tumour using RGS in five out of 17 cases ^23^. This may be partially due to the high threshold set by Hodolič, which at 4:1 would give a false negative result for many of the lesions found by Adams who used 2:1.

The alternative to using a fixed TBR is to look at the standard deviation of the count rate in normal tissues adjacent to suspected lesions. If, after multiple counts, the count rate from the target tissue is found to be more than 2 standard deviations higher than the count from the adjacent tissue, the suspect lesion is considered to be positive for tumour tissue. Three of the remaining four teams using this technique found good results with Benjegård detecting 11 of 12 tumours using the probe, Hall identifying three tumours only by use of the probe, and Öhrvall identifying 91.4% of lesions with the probe compared to 56.7% with pre-operative SPECT imaging ^10,17,18^. Wängberg et al., however, had more mixed results, identifying in vivo 27 of 31 gastric and midgut carcinoid tumours with the probe with only one false positive result, but struggled with localisation of pancreatic tumours, finding only 1 of the 4 tumours in this site ^20^.

Only one of the authors looked to determine the most appropriate TBR. El Lakis et al. used the histology results of the resected lesions and TBR values recorded at the time of surgery to create a receiver operator characteristic (ROC) curve and hence to examine the effect of varying the threshold on the specificity and sensitivity of the procedure ^27^. They found that the area under the ROC curve was 0.72 and that when using a threshold of 2.5:1 the sensitivity was 90% and the specificity was 25%. If the threshold was increased to 16:1, the sensitivity fell to 54%, but the specificity increased to 81%. Other researchers found that the specificity was higher at a lower threshold. As discussed earlier, the results of Wängberg et al. and Öhvall et al. found a sensitivity of 86% whilst maintaining a specificity of 80% ^18,20^. This discrepancy may be partially due to the small numbers of patients involved (44 for El Lakis and 44 in total for Wängberg and Öhvall), but the differences in the protocols mean that direct comparisons of results should be made with caution.

### Surgical and Pre-surgical Protocols

Some, although not all, authors included details of non-standard surgical procedures which they used to increase the TBR and hence the likelihood of locating small tumours.

The presence of high levels of physiological uptake in the liver, spleen, kidneys, and gall bladder presents challenges in locating small or low-specific activity lesions in the abdominal cavity. Wängberg advised the surgeons to aim the probe away from organs with high levels of normal tracer uptake ^20^. Review papers by García-Tavalera and Gulec take this further, both suggesting the placement of wide malleable retractors below the liver and spleen in order to provide some shielding whilst searching the tumour bed and move the organs with high-physiological uptake away from the tumour bed ^37,38^. Hodolič mentions the use of small lead shields, but does not elaborate on their construction or size ^23^.

Other authors attempted to reduce the physiological uptake in preference to shielding. Hodolič administered perchlorate or Lugol’s solution to block thyroid and gastric uptake before administering ^99m^Tc labelled TOC and administered fluids and furosemide to reduce urinary tracer retention ^23^. Because OCT is excreted in bile, Adams, Öhrvall, and Hubalewska-Dydejczyk all recommend pre-operative bowel prep with laxatives for 24–48 h prior to surgery to aid clearance ^18,19,24^. As a more extreme precaution, Benevento administered ^125^I-octreotide after commencing the laparotomy, taking the opportunity to clamp the common bile duct, effectively preventing hepatic excretion into the bowel whilst searching for a mid-gut carcinoid ^34^.

## Clinical Outcomes of Radioguided Surgery

Of the 26 papers reviewed, only six included patient follow-up, as detailed in Table 5. Benevento and Hosoya both followed the progress of their patients for 3 years following surgery ^13,34^. The two patients, with a midgut carcinoid and gastric carcinoid, were found to have no recurrence on follow-up. Fettich followed the two gastrinoma patients, insulinoma patient and carcinoid patient for 3 months following surgery, and again report no recurrence ^26^. Hodolič followed a selection of patients in their study for 6–12 months post RGS, and reports that the four patients who had failed laparotomy demonstrated tumours on follow-up scintigraphy. Only five patients with successful surgical outcomes were followed up by Hodolič; of these, three showed no recurrence, one demonstrated partial recurrence, and the final one demonstrated abnormal uptake on SRS ^23^. Hall monitored disease progression for between 4 and 32 months post-operatively; at the end of the follow-up period, all patients had reduced symptoms ^10^. Finally, Maccauro followed-up patients for between 2 and 10 months with all biochemical and imaging results indicating stable disease ^22^.

**Table 5:** Follow-up durations’ post-radioguided surgery

Author	Ref	Patient	Tumour type	Follow-up period	Recurrence?
Benevento et al	^34^	1	Midgut carcinoid	3 years	No
Hosoya et al	^13^	3	Gastrinoma	3 years	No
Fettich et al	^26^	5	Gastrinoma	3 months	No
		6	Gastrinoma	3 months	No
		7	Insulinoma	3 months	No
		8	Carcinoid	3 months	No
Hodolič et al	^23^	9	Pancreatic Carcinoma	6–12 months	No
		10	Pancreatic carcinoma	6–12 months	Yes
		11	Midgut carcinoid	6–12 months	No
		12	Midgut carcinoid	6–12 months	No
		13a	Gastrinoma	6–12 months	Yes
		13b	Gastrinoma	6–12 months	No
Hall et al	^10^	14	Gastrinoma	23 months	No
		15	Gastrinoma	7 months	No
		16	Gastrinoma	14 months	No
		17	Gastrinoma	8 months	No
		18	Gastrinoma	7 months	No
		19	Gastrinoma	4 months	No
Maccauro et al	^22^	20	Midgut carcinoid	2–10 months	No
		21	Midgut carcinoid	2–10 months	No
		22	Midgut carcinoid	2–10 months	No
		23	Rectal carcinoid	2–10 months	No
		24	Gastric carcinoma	2–10 months	No

## Radiation Doses to Staff During RGS

Two teams have published the radiation doses imparted to the surgical team as a result of implementing RGS, both using ^68^Ga-DOTA-Peptides. Sadowski et al. found the average effective dose received by the surgeons ranged between 40 and 270 µSv per surgeon for the 14 cases described, with the mean dose being 132 µSv ^29^. This followed an administered activity of 185 MBq, with an average delay of 78 min between administration and operation. Kaemmerer et al. found similar results; at between 19 and 120 min after administration of 180 MBq of gallium-68, they measured the surgical team dose as between 85 and 140 µSv, with the mean dose being 108 µSv per procedure ^31^.

No team has published radiation doses when working with TOC or OCT; it is difficult to extrapolate the dose from the gamma-emitting tracers by looking at the positron emitter, owing to the different photon energies and half-lives.

## Areas Which Merit Further Investigation

Whilst the literature surveyed is encouraging, there remain gaps in the evidence base for the benefits of performing RGS for GEP-NETs.

Whilst most of the authors have found RGS useful in locating GEP-NET tissue and in increasing the number of tumours identified over conventional imaging and surgical techniques, the aim of radical surgery is to prolong survival and minimise symptoms from the tumour. More appropriate end-points for assessing whether RGS improves patient care would be overall survival and/or time to progression, but given the indolent nature of many of the tumours gathering this data will involve very long-term follow-up. Just as important is the lack of control groups for comparison; given the comparatively small numbers of patients who undergo surgery for GEP-NETs, it is likely to be challenging for a single centre to recruit sufficient patients to allow for any difference in clinical outcome to be observed without a lengthy recruitment window. Neither of these is insurmountable; collaboration between centres would allow for more rapid recruitment, and surrogates for overall survival—for example biochemical progression—may allow for any potential benefits in clinical outcomes to be identified, whilst longer-term follow-up is in progress.

Whilst collaboration between centres would aid recruitment, it would be essential for a uniform RGS protocol to be adopted, and as yet the optimal protocol for RGS for GEP-NETs is yet to be determined. The work by El Lakis et al. to determine the most appropriate TBR threshold has allowed researchers to look at the effect on sensitivity and specificity of varying the TBR threshold and is the largest trial to date with 44 patients. However, the researchers used ^68^Ga-DOTA-peptides for the surgery, and it is not clear how well the results would convert to a gamma-emitting radiopharmaceutical in centres where technetium-99m or indium-111 labelled somatostatin analogues are used instead.

The researchers who used TOC have used a range of administered activities, and the time between administration and surgery varied from immediately post administration to a 24-h delay.

No reports clarify whether the patients were administered with “cold” somatostatin analogues such as octreotide or lanreotide, either as a maintenance treatment or as a pre-operative dose to minimise the risk of carcinoid crisis occurring during the operation. With many radiopharmaceuticals, administering a dose of a non-radioactive version of the tracer can “block” the relevant uptake mechanism. With the comparatively high mass of peptide administered in therapeutic doses, there is a risk that this may block the somatostatin receptors on the tumour cells, reducing the TBR value and making detection more difficult. The mass of the radiolabelled peptide administered is not stated in most papers, but those which have stated the dose used between 10 and 20 µg. Work by Bernhardt et al. to determine the optimum peptide mass to administer for imaging in a mouse model suggests an optimum dose in man of 200 to 2000 µg when scaled by body weight, which may suggest that using more peptide in the administered radiopharmaceutical (whether radiolabelled or “cold”) may improve the results of RGS ^39^.

Quality improvements are not only linked to clinical outcomes, with the cost improvements afforded by new investigations and treatments playing an increasing role in commissioning. Whilst there are high-level economic parameters such as the quality adjusted life year, or “QALY”, which can be used to assign a monetary value to the outcomes of a clinical procedure ^40^, simpler methods of measuring cost effectiveness can also be assessed. The cost of surgical resection not only is linked to the duration of the procedure and the running costs of the theatre, but also includes the costs of frozen section or other analysis of pathological samples; any necessary fluids, including blood and blood products which are at a premium in healthcare; recovery time, including time in the recovery room, high dependency units, general wards, and in the community; and the cost of treatment of any complications or side effects of the surgery or following treatment. Whilst Wang et al. included some of these factors in their definitions of success, they did not make explicit to what extent time and financial efficiencies were presented by the use of RGS ^12^. Without this information, it is difficult to determine what non-clinical benefits are presented by adopting RGS as routine practice.

Finally, the small numbers of patients who have undergone RGS means that it is difficult to tell which tracer is the most appropriate; whether the reduction in background scatter from the lower energy photons of technetium-99m tracers such as TOC outweigh the increase in TBR afforded by the longer half-life of OCT, or whether the increasing availability of the gallium-68 generators used in the manufacture of ^68^Ga-DOTA-peptides will lead to reductions in cost which will make them the tracer of choice.

## Limitations of the Literature Review

Although this review was performed largely following the PRISMA guidance, only one reviewer assessed the papers against the inclusion criteria. This may have resulted in some papers which were worthy of inclusion being missed or, less likely, inclusion of papers which did not meet the criteria. Given the limited literature on the use of RGS in GEP-NETs and the use of the PRISMA methodology, the impact of this on the quality of the review will have been minimal. In addition, as described in the methods section, the authors did not assess the included papers for quality or for risk of bias. This decision was taken as the papers included are largely case reports or report outcomes for small numbers of patients, and with no control element for comparison, resulting in low-quality evidence. The need for higher quality studies to support the weak evidence has been included in the text.

## Conclusions

Radioguided surgery appears to aid localisation of NETs in the gastrointestinal tract, with more equivocal evidence of benefit when applied to surgery for pNETs. The literature reports that RGS results in localisation of more tumours, assists the surgeon in discriminating between tumour and healthy tissue, and, in some reports, shortens the procedure. The evidence for improvements in clinical outcomes beyond this is still limited; however, in cases where localisation of tumours is likely to prove challenging, or where better discrimination may result in changes to surgical approach, RGS may be useful tool.

In the literature, most successful procedures employed a somatostatin analogue labelled with either indium-111, technetium-99m, or gallium-68. All three tracers have individual limitations and strengths, and the choice of which tracer to use will depend partly on local availability and experience. The duration of the interval between administration of the tracer and the start of the procedure is guided by the physical half-life of the tracer, with short half-life gallium-68-DOTA-peptides requiring administration in the hours immediately before the procedure, technetium-99m labelled tracers being administered up to 24 h prior, and indium-111 octreotide several days in advance.

The administered activity will also depend on the interval between administration and the procedure and will need to be optimised to that ensure sufficient tracer is retained in the patient at the time of surgery whilst minimising the radiation dose. The sensitivity of the probe to be used must also be considered, but in general, activities of 100–300 MBq of indium-111 at 24 h prior, 500–700 MBq of technetium-99m at 4–24 h prior, or 185 MBq of gallium-68 at up to 2 h prior to surgery have been successful.

Discrimination between tumour and healthy tissue can be made by applying either a set threshold ratio or by using a statistical technique. The ratio technique is the simplest to implement, but works best in a low background tissue such as the small bowel. For tumours in higher background areas, such as the liver or spleen, the statistical approach may prove to be more robust.

There is little additional patient preparation required above that for conventional surgical techniques—for longer lived indium-111 or technetium-99m tracers, prior treatment with laxatives may reduce the presence of excreted tracer in the gut, and ensuring the patient is well hydrated will aid clearance of the tracer from the blood and reduce the radiation dose to the kidneys. Prior treatment with a non-radioactive somatostatin analogue appears not to prevent tracer uptake and where appropriate for clinical reasons should not be considered a contraindication. Finally, radiation protection precautions must be considered, including assessment of the radiation dose to the surgical team performing the procedure. The staff dose from a single procedure is unlikely to be significant, but repeated use of the technique may result in radiation doses approaching national limits. Local expertise in radiation protection should be sought to optimise the radiation doses to both the patient and staff and to ensure compliance with relevant radiation protection regulations.

## Abbreviations

µg: Microgram
µSv: Microsievert
CT: Computed tomography
GBq: Gigabecquerel
GEP-NETs: Gastroenteropancreatic neuroendocrine tumours
keV: Kilo electronvolts
MBq: Megabecquerels
MGC: Mid-gut carcinomas
mIBG: Meta-iodobenzylguanidine
MRI: Magnetic resonance imaging
mSv: Millisieverts
NEC: Neuroendocrine carcinoma
NETs: Neuroendocrine tumours
OCT: OctreoScan, or indium-111 pentetreotide
PET/CT: Positron emission tomography with CT
PRISMA: Preferred reporting items for systematic reviews and meta-analyses
QALY: Quality adjusted life year
RGS: Radioguided surgery
ROC: Receiver-operator characteristic
SRS: Somatostatin receptor scintigraphy
SSA: Somatostatin analogues
SSTR: Somatostatin receptors
TATE: ^99m^Tc-EDDA/HYNIC-octreotate
TBR: Target to background ratio
TOC: ^99m^Tc-EDDA/HYNIC-TOC (Tektrotyd)
